# Light-Driven Charge Transport and Optical Sensing in Molecular Junctions

**DOI:** 10.3390/nano12040698

**Published:** 2022-02-19

**Authors:** Chaolong Tang, Mehrdad Shiri, Haixin Zhang, Ridwan Tobi Ayinla, Kun Wang

**Affiliations:** 1 Department of Physics and Astronomy, Mississippi State University, Mississippi State, MS 39762, USA; ct1772@msstate.edu (C.T.); ms4392@msstate.edu (M.S.); hz217@msstate.edu (H.Z.); 2 Department of Chemistry, Mississippi State University, Mississippi State, MS 39762, USA; rta79@msstate.edu

**Keywords:** molecular junctions, optoelectronics, photoswitch, photoemission, plasmonics, Raman sensing

## Abstract

Probing charge and energy transport in molecular junctions (MJs) has not only enabled a fundamental understanding of quantum transport at the atomic and molecular scale, but it also holds significant promise for the development of molecular-scale electronic devices. Recent years have witnessed a rapidly growing interest in understanding light-matter interactions in illuminated MJs. These studies have profoundly deepened our knowledge of the structure–property relations of various molecular materials and paved critical pathways towards utilizing single molecules in future optoelectronics applications. In this article, we survey recent progress in investigating light-driven charge transport in MJs, including junctions composed of a single molecule and self-assembled monolayers (SAMs) of molecules, and new opportunities in optical sensing at the single-molecule level. We focus our attention on describing the experimental design, key phenomena, and the underlying mechanisms. Specifically, topics presented include light-assisted charge transport, photoswitch, and photoemission in MJs. Emerging Raman sensing in MJs is also discussed. Finally, outstanding challenges are explored, and future perspectives in the field are provided.

## 1. Introduction

Following the original idea of adopting individual molecules as functional elements or interconnects in electrical circuits proposed by Aviram and Ratner [1], the past two decades have witnessed significant progress in both experimental and theoretical studies of molecular electronics (ME). These multidisciplinary efforts harnessing expertise from physics, chemistry, biology, and nanoengineering have transformed this research area into a new playground for exploring intriguing charge transport and energy conversion phenomena that are only accessible at the molecular scale. In particular, inordinate amounts of attention have been given to understanding charge transport through a molecular junction (MJ), a nanostructure in which individual molecules are linked to two conductive electrodes, and to develop strategies that can effectively control electron transmission through molecules [2,3,4,5,6,7,8,9,10,11,12,13,14].

Thanks to the rapid development of experimental techniques in MJ fabrications and measurements, researchers are now able to expand their investigations beyond pure charge transport. Many interesting transport phenomena in MJs have been reported to date, including the transistor (or gating) effect [15,16,17,18], conductance switching [19,20,21], thermal [22,23], thermoelectricity [24,25], photoswitching [26,27,28] and spintronics [29,30,31,32,33]. While most of these topics have been previously reviewed [34,35,36], we, in this work, will focus our attention on the optoelectronic phenomena in MJs, a topic that has been rarely surveyed in detail yet is rather important for furthering the development of the field. Light-matter interactions in illuminated MJs have drawn increasing amounts of attention in recent years due to many exciting potential applications associated with them, such as photovoltaics [37,38], organic light-emitting diode [39,40,41], chemical/bio-detection [42,43,44,45], and plasmonics [46,47,48]. Light, as an external stimulus for MJs, has several advantages over other external stimuli owing to its remote access, ultra-fast response time, a wide range of choices in wavelengths (or energy), and noninvasiveness. In an illuminated MJ, new transport effects may emerge due to various interactions, including light-molecule interaction, light-metal electrode interaction, and light-induced variation of the local environment, such as heating, generation of surface plasmon, and high electric field [49]. These unique interactions have led to the observation of a variety of phenomena, such as increases in molecular conductance, generation of highly energetic hot-carriers, photoemission, and molecular structure variations [50,51,52,53]. It is therefore highly expected that developing experimental approaches to control these interactions in illuminated MJs are essential for the design and production of next-generation molecular-scale optoelectronic devices.

To go beyond simple electrical measurements, powerful new experimental methods have been developed to reveal the chemical information of the molecular core in MJs, such as bond rotation, vibration, and rupture, which are usually not accessible in conventional single-molecule conductance measurements. This has been achieved via integrating photo-detection techniques with MJ fabrication platforms, such as combining break junction measurements with tip-enhanced Raman spectroscopy (TERS) [54,55,56,57] or mechanically controllable break junction surface-enhanced Raman spectroscopy (MCBJ-SERS) [58]. These emerging approaches have successfully enabled simultaneous characterization of electrical conductance and chemical structure information with single-molecule sensitivity and ultrahigh spatial resolution. In addition, involving plasmonic nanostructures in an MJ introduces an additional dimension of physical control and detection. Through monitoring plasmon resonance shifts and/or the change in molecular conductance, one can now capture a more complete picture of the fundamental mechanisms of charge migration in both the nanostructures and the molecules.

**Figure 1:**
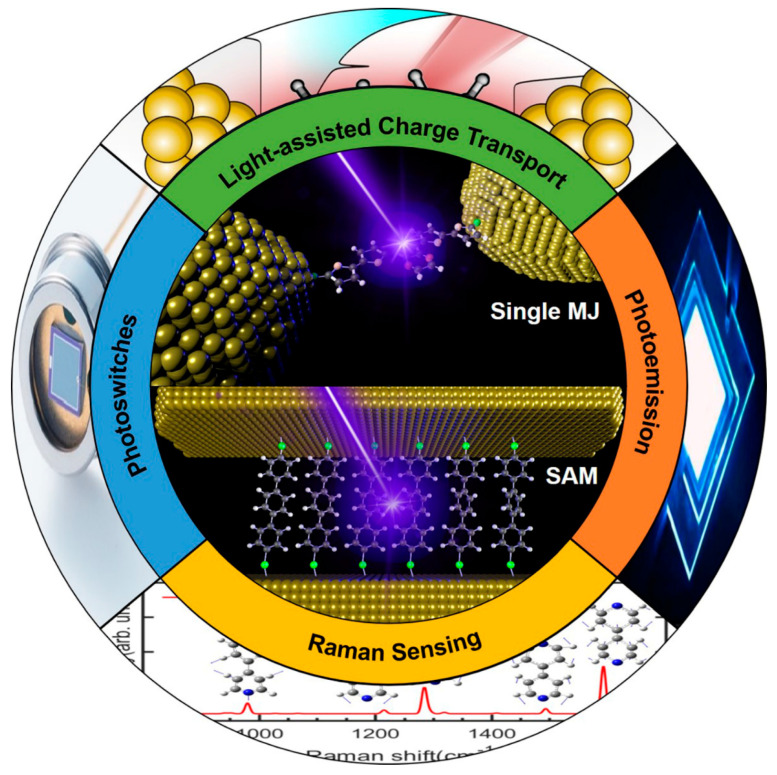
Schematic overview of the topics covered in this review.

To provide a detailed survey of these emerging optoelectronic phenomena in illuminated MJs, we present recent developments and progress of light-driven charge transport and optical sensing in MJs, including single MJs and self-assembled monolayer (SAM) junctions. The rest of the paper is structured as follows: first, we introduce advances in studying the optoelectronic effect in MJs, including the observation and understanding of photo-conductance enhancement, photoswitching, and photoemission; then we demonstrate recent progress in the emerging Raman sensing in MJs; lastly, we discuss outstanding challenges and new opportunities associated with these topics (Figure 1).

## 2. Light-Driven Charge Transport in MJs

Controlling charge transport through molecules by optical means has become a key approach in molecular electronics in the past decade. Molecular junctions excited by external light have been recently investigated in various experimental setups and simulated by different theoretical models. These studies aim to provide a fundamental understanding of charge transport phenomena associated with photon irradiation. In this section, we concentrate on discussing several key transport behaviors observed in illuminated MJs, including light-driven conductance enhancement, photoswitching, and photoemission.

### 2.1. Conductance Enhancement in Illuminated Single-MJs

Photon-assisted charge transport in tunnel junctions composed of single molecules, namely a change in molecular junction conductance under illumination, has been observed in distinct molecular systems. In these measurements, the conductance of single MJs was performed using single-molecule break junction techniques, such as the scanning tunneling microscopy break junction (STM-BJ) and mechanically controllable break junction (MCBJ) techniques. The heart of these break junction techniques is that single molecules are repeatedly linked between two metal electrodes via chemical anchoring groups modified at the two terminals of the target molecule. Junction formation is mechanically facilitated by a piezo transducer controlled by computer programs with subnanometer precision. To account for the variability of molecular conformation and contact geometry in break junction measurements, researchers usually construct histograms composed of thousands of individual conductance vs. time traces to obtain the peak value of the conductance histogram which is also considered as the most probable conductance of a single molecule. More details about the break junction techniques can be found in previous reviews [59,60].

#### 2.1.1. Photo-Induced Conductance Mechanisms in Single MJs

**Figure 2:**
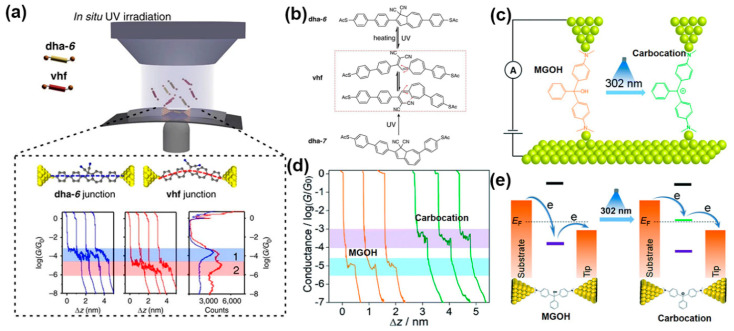
(**a**) Upper: Schematic of MCBJ test platform during the photo-conversion of dihydroazulene (dha-6) under in situ UV irradiation. The solution contains dha-6 and vinylheptafulvene (vhf) molecules. Lower: representative individual conductance-distance traces acquired using break junction measurements, blue for the dha-6 junction and red for the vhf junction. The junction bias is 100 mV. (**b**) The molecular structures and the conversion processes studied in the junction. Reprinted with permission from ref. [49]. Copyright 2017, Nature Publishing Group. (**c**) Scheme of the STM-BJ setup of MGOH molecule and photo-induced carbocation forms. (**d**) Typical conductance-distance traces of MGOH (orange) and carbocation (green) were recorded with STM-BJ measurements. (**e**) Schematic band diagram showing the position of the DFT resonances for carbocations respective to the two electrodes before and after light illumination. Reprinted with permission from ref. [65]. Copyright 2020, The Royal Society of Chemistry.

By comparing single-molecule conductance measured with and without light illumination in a break junction setup, conductance increase has been observed in various molecular species. Several distinct transport mechanisms were proposed to account for the observed conductance enhancement as detailed below:

*Photo-induced molecular structure change*. Light illumination on photochromic molecules and their derivatives, like dihydroazulene and triphenylmethane leuco, is known to alter their structures [61,62,63,64]. Several recent works have shown that the conductance of molecular junctions based on these photochromic molecules can be changed accordingly upon light illumination. Huang et al. employed the MCBJ technique coupled with UV irradiation (365 nm) to study the photo-thermal reaction processes in a photochromic dihydroazulene(dha-6)/vinylheptafulvene(vhf) system (Figure 2a) [49]. In their study, they performed conductance measurements by UV irradiation and heating treatments. It was found that the conductance of the initial dha-6 junction dropped by more than one order of magnitude, which was attributed to the fact that the dha-6 molecule underwent a ring-opening process of the five-membered ring under UV irradiation. By applying a 60 °C heat treatment for 30 min, the vhf can fully switch back to dha-6 through a ring-closing process (Figure 2b). Their approach could be used for probing the reaction kinetics, reversibility, and the occurrence of isomerization during the chemical reaction in MJs. In another recent study, using the STM-BJ technique, Bei et al. demonstrated photo-induced carbocation-enhanced charge transport in an MJ composed of malachite green leuco hydroxide (MGOH) [65]. As shown in Figure 2c, in the presence of 302 nm UV light, the MGOH produces malachite green carbocations, thus inducing a minor structural change where the orbital hybridization of the central carbon atom changes from initial sp^3^ to sp^2^. This structural change resulted in a large enhancement in single-molecule conductance by a factor of 34 (Figure 2d). It was found that both narrowing of the HOMO-LUMO gap and the enhancement of the transmission close to the Fermi levels contributed to the photo-induced carbocation-enhanced charge transport in MGOH when the carbocation formed (Figure 2e).

*Creation of new conduction channels under illumination*. Recent studies have also shown that new transmission channels can be created for electrons when an MJ is exposed to light excitation. A typical example is a porphyrin-C_60_ dyad molecule consisting of a porphyrin chromophore and a C_60_ electron acceptor [66]. Specifically, when studied in indium tin oxide (ITO)-gold junctions using the STM-BJ technique, this complex was found to generate a charge-separated state when illuminated with a 520 nm laser [67]. It was found that the fraction of molecules in the high conductance state increased linearly with laser power density up to a maximum of 50% at 200 mW/cm^2^ (the threshold for photodamage). Further transient absorption spectra showed only dyad molecule on ITO surface to form a long-lived charge-separated state instead of in solution or in films of the porphyrin alone. Therefore, absorption of light led to the formation of P^●+^−C_60_^●−^ by photo-induced electron transfer, which further resulted in charge migration away from the site of initial electron transfer, through hopping to adjacent molecules and/or migration into the ITO conductive substrate. It was noted that, although the exact nature of charge states was not clear, the ITO substrate facilitated the charge extraction from the photoexcitation.

**Figure 3:**
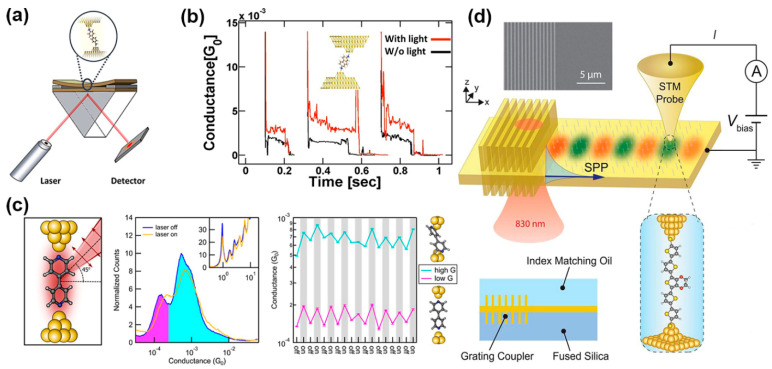
(**a**) Squeezable break junction setup for single-molecule conductance measurements. (**b**) Corresponding data plot of the conductance of 2,7-diaminofluorene single MJs enhanced upon p-polarized laser irradiation due to the plasmon-induced oscillating field within the nanoscale metal gap of the junctions. Reprinted with permission from ref. [68]. Copyright 2013, American Chemical Society. (**c**) Schematic of an illuminated metal-molecule-metal junction with 4,4′-bipyridine (BP) in the low-conducting geometry (left panel). Arrows indicate the direction of polarization; Logarithmically binned 1D conductance histograms of BP junctions at 180 mV bias for dark (laser off) and illuminated (laser on) junctions (middle panel); A series of conductance measurements where the laser was successively turned on and off (right panel). Reprinted with permission from ref. [69]. Copyright 2017, American Chemical Society. (**d**) Schematic of the experimental setup used to map hot-carrier energy distribution. Single MJs were formed between a grounded Au film with an integrated grating coupler and a biased Au STM probe. SPPs were excited by illuminating the grating coupler with an 830-nm continuous-wave laser. Reprinted with permission from ref. [51]. Copyright 2020, American Association for the Advancement of Science.

*Photo-assisted tunneling and generation of hot-carriers*. Under this mechanism, the conductance enhancement in illuminated MJs can result from either a plasmon-induced electric field within the nanogap of the junction [68], as described by the Tien–Gordon model in photon-assisted tunneling (PAT), or from the generation of hot electrons [69]. Surface plasmons are capable of concentrating light into the deep subwavelength between metallic nanogaps and consequently of giving rise to electromagnetic field enhancement. This plasmon coupling to the molecule can modulate the conductance in single MJs. With the generation of surface plasmons, a rectified dc current was formed and observed in MJs, and surface plasmons were shown to be the optical origin of causing conductance enhancement [70,71]. In detail, Vadai et al. carried out a conductance measurement on a 2,7-diaminofluorene (DAF) single MJ by using the squeezable break junction (SBJ) technique under laser irradiation (781 nm) [68]. SBJ was made of two Au-covered glass slides with an initial gap of 500 nm between them. The gap was precisely controlled with high accuracy to form single-atom contacts by applying compressive or tensile strain on the top slide (Figure 3a). Similar to MCBJ and STM-BJ, the conductance traces were recorded once the top surface touched the bottom slide. Moreover, the Au surfaces were sufficiently rough so that surface plasmons can be created within the nanogap. The results displayed that only p-polarized illumination produced surface plasmons within the gap and thus resulted in a conductance increase (Figure 3b). In contrast, s-polarized irradiation did not affect the conductance behavior of the junctions. Given that the HOMO-LUMO energy gap of DAF (>3 eV) is much larger than the energy of the generated plasmons (1.59 eV), the conductance increase was predominantly contributed by the plasmon-induced oscillating electric field. The total energy for an electron through a tunneling junction becomes *E* ± *ℏω*, implying that the initial electron with energy *E* can absorb or emit photons with energy of *ℏω* to complete the tunneling process. Therefore, the transmission probability and conductance of a junction were enhanced. However, the photon-assisted transport process is diabatic in the Tien–Gordon model, which assumes that the interactions between the electrons and the electromagnetic field occur only in the molecular bridge rather than on the electrodes. A recent study argued that hot electron transport constituted most of the light-induced enhancement of conductance in single 4,4′-bipyridine MJs when electrons in the electrodes absorbed photons (980 nm) and that hot electrons’ relaxation time can be long (Figure 3c) [69]. The Tien–Gordon model would be appropriate to describe the photon-assisted transport only if the lifetime of hot electrons was shorter than the charge transfer time or no light absorption occurred on the electrodes. Although plasmonic nanostructures have been extensively studied in nanophotonics to generate strongly localized surface plasmons, there were few attempts to incorporate these nanostructures into MJ setups. Only very recently, such a junction configuration containing a gold thin-film plasmonic nanosurface as the bottom electrode was realized by Reddy et al. [51]. In this work, single molecules with a sharp transmission resonance close to the gold Fermi level were trapped between an ultrathin (6 nm) gold film supporting surface plasmon polaritons (SPPs) and a gold STM tip (Figure 3d). Upon illumination from an 830 nm laser (resonant wavelength) of the grating coupler fabricated on the thin-film substrate, propagating surface plasmons were launched. Consequently, a noticeable enhancement in single MJ current was observed due to the plasmonic excitation, which was shown to be strongly polarization-dependent. Further analysis revealed that Landau damping is the dominant physical mechanism of hot-carrier generation at the junction. This new experimental approach could open avenues for future investigations of various nanophotonic and plasmonic devices.

**Figure 4:**
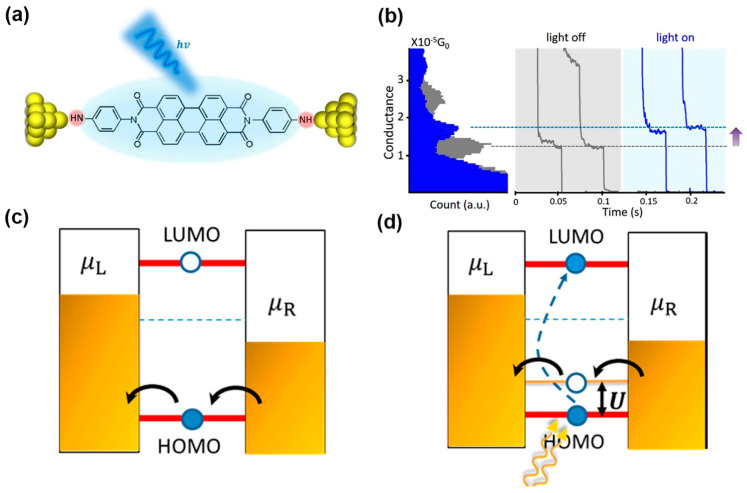
(**a**) Schematic of the STM-BJ setup, an NH_2_-PTCDI-NH_2_ molecule linked to two electrodes and illuminated with laser light. (**b**) The 1D conductance histograms in the dark (gray) and under illumination (blue). Right panels are typical conductance traces. (**c**) In the dark, the current is dominated by hole transport through the HOMO. (**d**) The LUMO is partially filled with light illumination, resulting in a hole entering the HOMO, and hence lifting the HOMO level toward the Fermi level to increase conductance. Reprinted with permission from ref. [72]. Copyright 2018, American Chemical Society.

*Exciton-binding within the molecule*. In recent work, Zhou et al. demonstrated that an exciton-binding effect, i.e., the generation of an electron-hole pair in the molecular bridge upon laser illumination, can occur in symmetric single MJs [72]. Specifically, the STM-BJ technique was employed to trap an NH_2_-perylene tetracarboxylic diimide (PTCDI) -NH_2_ molecule to two gold electrodes via Au-amine bonds (Figure 4a). The conductance of PTCDI molecules showed a significant and reversible change when the MJ was measured in the dark and under the illumination of a 495 nm laser. It is worth noting that the incident laser had an energy in resonance with the HOMO-LUMO gap of the PTCDI molecule (Figure 4b). In such a junction system, the most likely transport model through an MJ was the following: under resonant illumination, electrons were excited from the HOMO to the LUMO, which led to an intramolecular Coulomb interaction between the electrons and holes. This effect in turn resulted in that the HOMO (the frontier orbital) level of the molecule was effectively shifted closer towards the Fermi level, increasing the junction conductance (Figure 4c,d). This simple design could find use in optimizing the performance of future molecular switches.

#### 2.1.2. Illuminated Metal-Molecule-Semiconductor MJs

**Figure 5:**
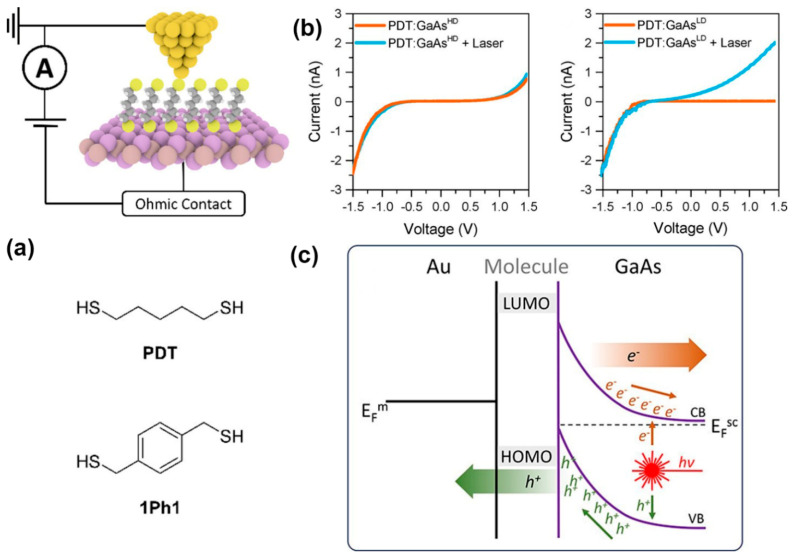
(**a**) Schematic diagram of the MMS junction and the molecules used for the study. (**b**) Junction current-voltage characteristics for (**left**) PDT on highly doped GaAs, (**right**) PDT on lower doped GaAs. (**c**) Schematic band diagram for the illuminated MMS junction under reverse bias. Reprinted with permission from ref. [76]. Copyright 2017, American Chemical Society.

Noble metals have been widely used as contact electrode material for molecular junctions due to their ohmic contact electrical behavior, being insensitive to oxidation and tarnishing, and the availability of making large-scale devices [73,74,75]. However, for a metal-molecule-semiconductor (MMS) junction, the semiconducting electrodes may increase the functionalities of the MMS junction by displaying its rectifying behavior and the occurrence of spontaneous photocurrent. The optoelectronic behavior of MMS junctions has also been investigated recently. Employing the STM-BJ technique, Vezzoli et al. studied the Au-1,5-Pentanedithiol (PDT)-GaAs junction and Au-1,4-Phenylene- (dimethanethiol) (1Ph1)-GaAs junction under laser irradiation, respectively (Figure 5a) [76]. They found that junctions with lower doped GaAs gave high rectification ratios in the dark and a high photocurrent under reverse bias. In contrast, junctions with highly doped GaAs presented poor rectification in the dark and a low photocurrent (Figure 5b). This difference was attributed to the thicker depletion layer or space charge region in lower doped GaAs. The thicker layer served as a wider tunneling barrier which not only prevented electron transport from the metal to the lower doped GaAs when junction bias was lower than the Schottky breakdown voltage but also increased the number of electrons and holes generated in the conduction band and valence band of GaAs under laser illumination. Band bending at the MMS junction separated the photogenerated electrons and holes. The electric field in the space charge region pushed photogenerated holes to the molecule, while the photogenerated electrons were driven to the GaAs electrode. Thus, photocurrent is produced under reverse bias (Figure 5c). Furthermore, the radius of the hemispherical space charge region was enlarged as the reverse bias potential was increased. The fraction of the illuminated region that contributed charge carriers to the photocurrent also increased.

### 2.2. Photo-Induced Conductance Switching in Single MJs

The ability to reliably switch the conductance of a single molecule between two or more distinct states is pivotal for the use of molecules as building blocks in future optoelectronics, computing, and chemical/bio-sensing applications. Conductance switching effects have been demonstrated in MJs exposed to various external stimuli, such as electric field [77,78,79], mechanical modulation [80], chemical [80], and optical means [81]. Here, we focus specifically on the conductance switching behavior in MJs driven by optical excitation.

#### 2.2.1. Photoswitches Based on Diarylethene Molecules

**Figure 6:**
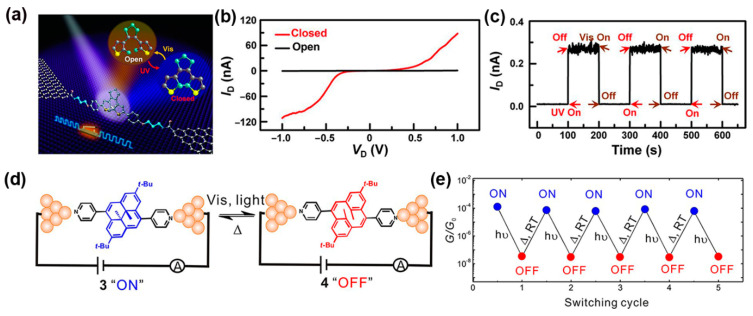
(**a**) Schematic of a graphene-diarylethene-graphene junction. (**b**) I-V curves of diarylethene molecule in open (black line) and closed (red line) state. (**c**) Real-time current through a diarylethene MJ upon exposure to ultraviolet (UV) and visible (Vis) radiation switches. Reprinted with permission from ref. [11]. Copyright 2016, American Association for the Advancement of Science. (**d**) The reversible structure changes induced by isomerization of a single bispyridine-substituted DHP molecule to CPD molecule in MCBJ layout. The closed form DHP (“ON”) with higher conductance than the open form of CPD isomer (“OFF”). (**e**) Five sequential fully reversible cycles of the photothermally triggered in situ conductance switching between DHP (“ON”) and CPD (“OFF”). Reprinted with permission from ref. [93]. Copyright 2013, American Chemical Society.

The diarylethene molecule has been found to exhibit a strong photochromic effect: it can undergo a structural transition from open-ring to closed-ring under UV irradiation, and its closed-ring isomer can be switched back to the open-ring form when exposed to visible light [82,83]. Thanks to the excellent thermal stability of both isomers, fast photoresponse, and high reversibility, diarylethene and its derivatives have been broadly researched for their potential in optical switching applications [84,85]. In general, the open-ring form turned out to have a lower conductance than that of the closed-ring. In a recent work by Jia et al. [11], the diarylethene molecule was investigated in a graphene-diarylethene-graphene junction where the molecule is covalently connected to two graphene electrodes. Under such a setup, it was observed that the closed-ring form of diarylethene could not be isomerized back to the less-conducting open form because the molecule-electrode coupling quenched the excited state of the nonconjugated isomers in the open-ring form, and the energy was transferred from the molecule to the carbon electrodes. To overcome this quenching effect, they incorporated three methylene (CH_2_) groups onto the two ends of the diarylethene molecular backbone to reduce the molecule-electrode coupling. Impressively, this modified diarylethene with graphene electrodes can be reversibly switched between two forms in a rather robust and reproducible manner (Figure 6a). Further theoretical analysis indicated that the HOMO of diarylethene entered the junction transmission window in the applied bias range, therefore enabling conduction. The current-voltage (I-V) characteristics of the single MJs are shown in Figure 6b. The onset of the current through the closed form molecule occurred at a junction bias of ~0.3 V, whereas no considerable conduction through the open molecule was observed up to 1 V. Notably, very remarkable accuracy (on/off ratio of ~100), stability (over a year), and reproducibility (46 devices with more than 100 cycles) were successfully demonstrated in this work (Figure 6c), paving a critical path towards the development of real-world applicable molecular switches.

#### 2.2.2. Photoswitches Based on Azobenzene Molecules

Azobenzene, another popular photochromic molecule, adopts *trans* and *cis* conformations. Similar to diarylethene, *trans*-to-*cis* isomerization of azobenzene occurs upon UV light irradiation. Reversely, the *cis*-to-*trans* conversion happens when the molecule is exposed to visible light. Additionally, the *cis*-to-*trans* isomerization process can also be triggered by thermal activation [86,87]. It has been reported that *trans* isomers usually have a lower conductance than their *cis* isomers [88]. For example, Meng et al. recently studied a new azobenzene derivative, 2′-p-tolyldiazenyl-1,1′:4,4′-terphenyl-4,4″-dicarboxylic acid (TTDA) in a graphene-molecule-graphene junction [89]. They covalently incorporated individual TTDA molecules into graphene electrodes through amide bonds to form single MJs. In this case, a conjugated aromatic chain consisting of an azobenzene side-group as the molecular bridge was chosen, since the isomerization of azobenzene incorporated via side substitution does not essentially alter the geometric structure of the charge transport pathway. Compared to the isomerization groups placed in the backbone, the azobenzene side-group increased the stability of molecular junctions originating in the length change during isomerization. The photoswitch effect at low bias showed that the device can reversibly switch its conductance states between high and low with an on/off ratio of ~2.1 under sequential ultraviolet (UV) and visible light irradiations at a very small source-drain voltage of 10 mV. It is noteworthy that the on/off ratio of azobenzene is much lower than that of the diarylethene molecules, although switching occurs at a lower junction voltage. When *trans*-to-*cis* isomerization occurs, HOMO (the perturbed frontier orbital for azobenzene) moves towards the graphene Fermi level, thus resulting in a larger transmission around the Fermi level. As a result, the different forms of azobenzene (either *trans* or *cis*) may serve as an efficient chemical gate to modulate charge transport of the molecular main chain. The asymmetric spatial orientation of the azobenzene unit played a key role in the trans-cis isomerization process, which provides a new perspective to construct practical single-molecule devices and logic gates.

#### 2.2.3. Photoswitches Based on Dihydropyrene Molecules

Dimethyldihydropyrene (DHP) and cyclophanediene (CPD) are both photochromic isomers. DHP is a polycyclic π-conjugated unit and CPD is its less π-conjugated isomer. They can be reversibly converted between two conformations, i.e., the colored closed state DHP can be transited to the colorless open state CPD under visible light irradiation. Both visible light and thermal activation are able to revert the CPD isomer to the DHP molecule [90,91]. The polycyclic hydrocarbon skeleton of the DHP unit can be chemically functionalized in many ways for applications, such as optoelectronic materials or molecular devices [92,93]. Roldan et al. reported the first single-molecule conductance measurements with DHP/CPD isomers using the MCBJ technique [93]. Under Xe-Hg lamp light irradiation (λ ≥ 490 nm), the color of molecule 3 changed steadily from green to colorless as the photo-conversion of 3 into its corresponding open isomer 4 proceeded (Figure 6d). The reversible photoisomerization process was achieved by irradiation of a solution of 4 with UV light (254 nm) or thermal activation (Figure 6d). As shown in Figure 6e, the MCBJ measurements started with the closed form 3 (ON state), and its conversion to form 4 (OFF state) was completed with visible light illumination (≥490 nm). Next, the 3 (ON state) recovered from the 4 (OFF state) upon thermal activation in the dark. There was no attenuation in conductance of each state after five full switching cycles, resulting in an ON/OFF ratio of ~10^4^. This is a vivid indication that DHP derivatives are promising candidates for molecular optoelectronics applications.

Besides the abovementioned photochromic molecules, there are several other molecular species that present similar transport behavior [94,95,96]. For example, spiropyran was reported to show a transformation from the closed-ring SP isomer to the open-ring zwitterionic merocyanine (MC) isomer with light stimuli [94]. Furthermore, photoswitchable molecules, such as molecular wires consisting of oligophenyleneimine with photoswitchable dithienylethene linkers [82] and norbornadiene-quadricyclane isomers [95], have been investigated for exploring photo-induced charge transport mechanisms in single MJs. These recent studies on conventional photochromic molecules and many other emergent species are essential for optimizing molecular design.

### 2.3. Photoemission in MJs

**Figure 7:**
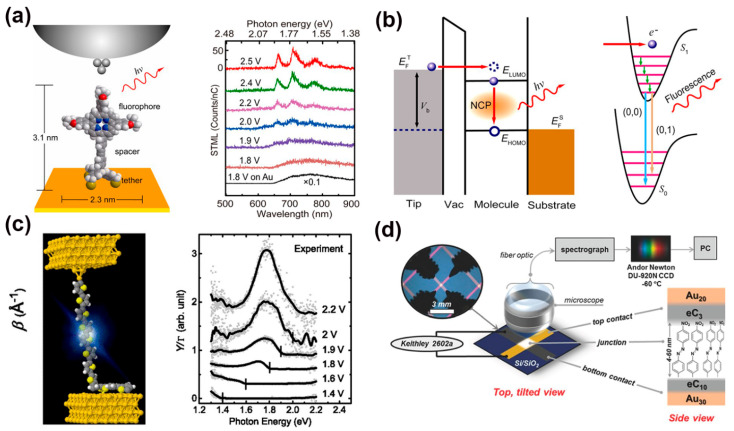
(**a**) Left: STM junction configuration of a self-decoupled porphyrin molecule on Au (111) surface and localized electrical excitation from a nanotip; Right: bias dependence of STM-induced luminescence spectra. (**b**) Energy band diagrams showing molecular excitation through the injection of hot electrons at positive sample bias, which is then followed by axial nanocavity plasmon emission and generation of molecular fluorescence via a Franck–Condon transition from the excited state (S_1_) to the ground state (S_0_). Reprinted with permission from ref. [100]. Copyright 2013, American Chemical Society. (**c**) Schematic view of a fluorescent polythiophene junction and its plasmon-corrected spectra of the light at different voltages. Reprinted with permission from Ref. [39]. Copyright 2014, American Physical Society. (**d**) Experimental setup for monitoring light emission from MJs consisting of 4–60 nm thick layers of organic molecules between conducting contacts. Reprinted with permission from ref. [106]. Copyright 2016, WILEY-VCH Verlag GmbH & Co. KGaA, Weinheim.

In recent years, luminescence, i.e., photoemission, from organic molecules on metals, has gained considerable interest for its potential applications in the display and lighting industries [97,98,99]. However, when molecules are directly adsorbed on or very close to metal surfaces, the intrinsic molecular fluorescence is often quenched by the strong interaction between the molecules and the substrate [100]. It is believed that decoupling the interaction between the molecular core and the electrode is key to retaining the molecular-specific photoemission. Therefore, the single-molecule electroluminescence phenomenon was predominantly observed in an STM setup with the molecule covalently bonded only to the metal substrate and not in direct contact with the STM tip. Using this setup, Zhu et al. designed a tripodal anchor porphyrin molecule that could produce molecular electroluminescence on the single-molecule scale [100]. The porphyrin moiety was proven effective in generating molecular electroluminescence in the STM junction [101], and the tripodal anchor not only acted as a decoupling spacer to minimize the molecule/electrode interaction but also made the tip-molecule-substrate junction highly asymmetric along the tip axis. This configuration was expected to offer the maximal emission enhancement due to the orientation of the molecular transition dipole favoring its coupling to the axial nanocavity plasmon (NCP) (Figure 7a). The STM-induced luminescence spectra evidenced that the peak position was nearly constant with the excitation voltage. A threshold voltage appeared at ~1.9 V, which equaled the optical bandgap of the molecule. Above the threshold voltage, the STM-induced luminescence intensity increased with the junction bias. It was suggested that the porphyrin molecule was excited by hot electron injection when both the HOMO and LUMO levels fall inside the electrochemical potential window defined by the bias voltage, and then the excited state decayed back to the ground state through Franck–Condon π*-π transitions (Figure 7b). This study may provide a new route to realize electrically-driven single-molecule light sources. In contrast to the aforementioned work in which the STM tip was not in contact with the molecule, Reecht et al. used the STM tip to partially lift a fluorescent polythiophene wire from an Au (111) surface [39]. In this setup, both ends of the conjugated wire were directly in contact with the gold electrodes, as shown in Figure 7c. A significant portion of the polymer wire was lifted up by the STM tip and suspended in the junction. Under such a junction configuration, light emission from the junction was observed when a positive sample bias was applied. Plasmon-corrected spectra revealed a broad resonance at 1.8 eV, whose maximum did not shift with voltage (larger than 2 V) (Figure 7c). This observation and detailed analysis based on a two-level Gaussian model suggested the asymmetric position of the HOMO-LUMO gap of the molecule with respect to the Fermi level and the asymmetric voltage drop repartition at the interfaces were major contributors to the emission properties of the junction. The similarity between this work and organic light-emitting diodes (OLED) provided a perspective towards single-molecular light-emitting components.

Photoemission detection in MJs also represents a powerful tool for determining charge transport mechanisms through molecules [102,103]. In this regard, Tefashe et al. recently investigated symmetric SAM junctions consisting of Ru- (bpy)_3_ oligomers of varying lengths sandwiched between conducting carbon contacts [104]. When the molecular length (or layer thickness d) was smaller than 4 nm, the current density vs. bias curves for MJs showed a strong exponential dependence on thickness, and light emission was weak, which was attributed to hot electrons traversing the molecular layer and emitting light in the top contact. Nevertheless, a bipolar charge injection mechanism became dominant when d > 4 nm and the junction bias was greater than 2.7 V. The light emission intensity unexpectedly increased in the range of 600–900 nm. Furthermore, light emission has been used to probe energy losses in MJs. Ivashenko et al. studied the transport of thin and thick aromatic layers in MJs by characterizing the light emission properties [105]. In their study, energy loss was observed to be strongly dependent on molecular layer structure and thickness for thicker molecule layers, which suggested that inelastic electron hopping is the dominant transport mechanism in thicker layers. For carbon-molecule-carbon molecular junctions containing aromatic molecular layers like anthraquinone, nitroazobenzene, naphthalene diimide, and bis-thienyl benzene with thicknesses d < 5 nm, charge transport was elastic, i.e., no energy loss was observed (Figure 7d) [106]. The maximum emitted photon energy was equal to eV_app_, where V_app_ was the bias applied across the molecular junction. For d > 5~7 nm, the transport mechanism changed from elastic to inelastic, and activationless transport up to 65 nm molecular layer thicknesses was seen. Therefore, probing light emission from SAM junctions proved to be a valuable method for understanding the transport mechanisms in MJs.

### 2.4. Optoelectronic in SAM Junctions

In addition to single MJ studies, SAM junctions featuring a great number of individual molecules connected in parallel represent another major focus for optoelectronic studies. Unlike obtaining conductance vs. displacement traces using the single-molecule break junction approach, researchers examine current density (*J*) vs. junction bias (*V*) as the key feature for understanding the transport behavior in SAM junctions.

#### 2.4.1. Photo-Induced Transport in SAM Junctions

**Figure 8:**
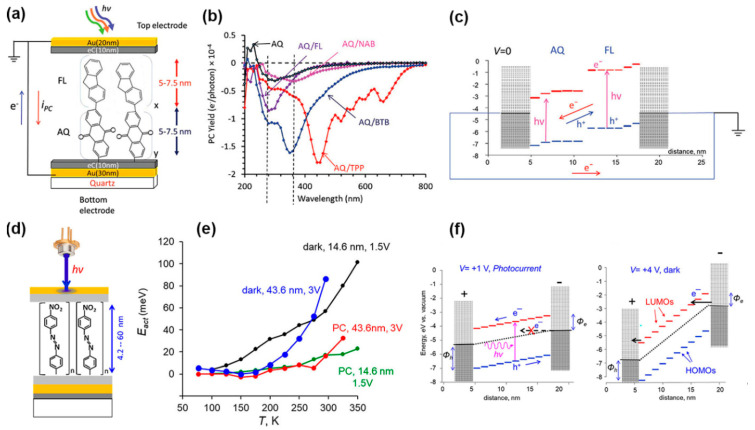
(**a**) Schematic of an Au_30_/eC_10_/AQ_6_/FL_6_/eC_10_/Au_20_ bilayer MJ structure. Where eC is the electron beam-deposited carbon contact. (**b**) Photocurrent yield for four 5–7.5 nm thick bilayer MJs having AQ as a first (bottom) layer and single-layer AQ. (**c**) A possible schematic mechanism for photocurrent production in an AQ/FL bilayer MJ at zero bias, with HOMO orbitals blue and LUMOs red. Reprinted with permission from ref. [109]. Copyright 2019, WILEY-VCH Verlag GmbH & Co. KGaA, Weinheim. (**d**) An illustration of Au_30_/eC_10_/nitroazobenzene/eC_10_/Au_20_ MJ with a molecular layer thickness from 4.2–60 nm. (**e**) Activation energies (E_act_) versus Temperature (T) for dark and photo-induced currents with two different molecular layer thicknesses and biases. (**f**) Energy band diagram of dark and photo-induced currents for nitroazobenzene between carbon electrodes. Φ*_h_* and Φ*_e_* are the hole and electron injection barriers, and the energy scale is referenced to vacuum. Reprinted with permission from ref. [110]. Copyright 2020, American Chemical Society.

In parallel to researching single molecules, researchers have also devoted considerable efforts to understanding photo-induced transport phenomena in SAM junctions. This is primarily because SAM junctions can be more easily integrated into the existing solid-state fabrication, and their thin-film devices may lead to applications in photo-detection, lighting, and energy harvesting. Recent studies in this direction have focused on exploring molecular species whose transport properties can be effectively tuned by light [107,108,109,110]. For example, Shailendra et al. [109] investigated Au/carbon/bilayer molecules/carbon/Au MJs consisting of two different 5–7 nm thick molecular layers between the identical top and bottom carbon contacts (electron beam-deposited carbon). They examined how the oligomers’ molecular orbitals and optical absorbance spectra affect the photocurrent response, the direction of photo-induced charge transport, and maximum response wavelength (Figure 8a). They found that the photocurrent spectrum closely follows the absorption spectrum of the molecular layer, suggesting electron-hole generation as the origin of the photocurrent. As is shown in Figure 8b, the organic/organic interface between the two molecular layers, such as anthraquinone (AQ)/fluorene (FL), AQ/nitroazobenzene (NAB), AQ/bis-thienyl benzene (BTB), and AQ/tetraphenyl (TPP), was the primary determinant of photocurrent polarity. In an unbiased bilayer AQ/FL MJ, the donor molecule (FL) produces an upward potential shift of both HOMO and LUMO energies. In contrast, the energies of the acceptor (AQ) are shifted downward. The combination of electrode-molecule and organic/organic interfaces generates an internal electric field that drives the photocurrent (Figure 8c). It is observed that when an external bias is applied, the photocurrent of the illuminated SAM junctions exceeds the dark current by a factor of 10^2^ to 10^5^, depending on the bias, bilayer structure, and wavelength, implying the SAM junctions as potential photodetectors. They found that an internal quantum efficiency of 0.14 electrons per absorbed photon can be achieved. The same group [110] further studied the bias and temperature dependence of both dark and photo-induced currents in nitroazobenzene SAM junctions with a layer thickness ranging from 4 to 60 nm (Figure 8d). It was shown that the dark current became weakly dependent on the thickness of nitroazobenzene and temperature in 15–60 nm thick MJs. Both dark and photo-induced currents manifested near-zero attenuation coefficients, and they were activationless transport (E_act_ < 5 meV) below ~200 K (Figure 8e). An orbital-mediated long-range transport model that accounts for the weak distance, bias, and temperature dependence of the photo-induced and dark currents is shown in Figure 8f. In this model, electrons in the negative electrode cannot transfer into empty LUMOs in the nitroazobenzene under a low bias of 1 V due to the large energy difference between the electrode Fermi level and frontier orbitals. Photoexcitation helps electrons overcome these barriers by generating electron-hole pairs, which can separate and move toward empty orbitals in the electrodes. The weakly interacting oligomeric subunits compose sequential small barriers and short tunneling distance. Electron transport between adjacent LUMOs could be considered resonant. Such transport is nearly activationless. In contrast, carriers must be injected into orbitals at a high bias to produce a large dark current possible with coherent tunneling. This new transport mechanism could increase the possible layer thickness accessible in molecular electronic devices.

#### 2.4.2. Photoswitches in SAM Junctions

**Figure 9:**
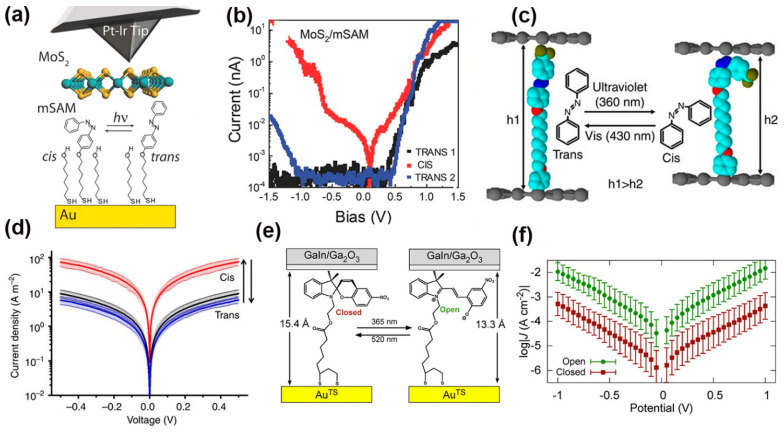
(**a**) Schematic diagram of the Au/azobenzene derivatives mixed-SAM/MoS_2_/Pt-Ir heterostructure junction measurement. (**b**) Typical I-V characteristics in a semilogarithmic scale for the MoS_2_/mixed-SAM heterostructure junction before (*trans*_1_), after UV (*cis*), and after white light exposure (*trans*_2_). Reprinted with permission from ref. [121]. Copyright 2015, WILEY-VCH Verlag GmbH & Co. KGaA, Weinheim. (**c**) Reversal conformational changes in aryl azobenzene molecules tunneling junction under light irradiation (h1 and h2 are the distances between the two graphene electrodes, respectively). (**d**) Current density–voltage plots in a log scale for the graphene/aryl azobenzene monolayer/graphene devices during light-induced isomerization. Reprinted with permission from ref. [122]. Copyright 2013, Nature Publishing Group. (**e**)Schematic of SAMs of spiropyran moiety in EGaIn/Ga_2_O_3_/SAM/Au junctions and light-induced isomerization between their open and closed forms. (**f**) Current density versus voltage plots of junctions in (**e**) the open (green) and closed (red) forms. Spiropyran moiety monolayers mixed with hexanethiol. Reprinted with permission from ref. [123]. Copyright 2016, American Chemical Society.

The photoswitch behavior of many photochromic molecules, such as azobenzene, spiropyran, diarylethene, and dithienylethene, have also been broadly investigated in the SAM junction setup [111,112,113,114,115,116,117,118,119,120]. To optimize the charge injection and extraction in electronic devices, recent efforts have been devoted to understanding the electronic properties of SAM junctions featuring SAMs sandwiched between metallic contacts and 2D semiconductor materials, such as 2D crystals. For instance, Margapoti et al. reported a photoswitchable diode effect in the 2D MoS_2_-azobenzene SAM heterostructures using the conductive-atomic force microscopy (c-AFM) technique (Figure 9a) [121]. In their study, the mixed-self-assembled monolayer (mSAM) consisting of the azobenzene derivative (4-(1-mercapto-6-hexyloxy)-azobenzene) and a spacer SAM 6-(2-mercapto)-1-hexanol was assembled on an Au substrate through chemisorption. The introduction of spacer avoided potential aggregation, which could impede the photo-mediated molecular conformational switching. A layer of MoS_2_ was directly exfoliated on top of the mSAM. The I-V characteristics in the semilogarithmic scale for monolayer-MoS_2_/mSAM heterostructure showed not only a photoswitch behavior but also a rectifying effect (with a rectification ratio up to ~10^4^) (Figure 9b). UV irradiating at 366 nm induced conformational isomerization of azobenzenes from *trans*-to-*cis*, which led to an abrupt transition from rectifying to symmetric I-V behavior. It was further demonstrated that the rectification effect can be recovered by overnight irradiation with white light. It was proposed that the photoswitchable transport characteristics originated from a large difference between *cis*-mSAM and *trans*-mSAM contact potentials. The rectification behavior was attributed to the strong misalignment of the *trans*-mSAM MoS_2_ Fermi level with the Au Fermi level. Furthermore, SAMs bonded to graphene electrodes could enable the development of highly stable, transparent, and flexible electronics. In this direction, Seo et al. investigated a photoswitchable aryl azobenzene monolayer with one side chemically self-assembled and the other side physically anchored between two graphene electrodes (Figure 9c) [122]. During the device fabrication process, the aryl alkane, or azobenzene monolayers were grafted onto the graphene bottom electrode for chemically forming SAM, and another layer of graphene was later incorporated into the SAM as the top contact. Current density plots of the aryl azobenzene monolayers exhibited two different conductance states with UV/Vis illumination. Similar to the observation in single MJs, the *trans*-isomer with low conductance can be transformed reversibly into a *cis*-isomer with high conductance. The higher conductance of the cis-isomer mainly originated from a relatively short tunneling distance (Figure 9d).

In addition, visible (520 nm) or UV (365 nm) light can covert a spiropyran moiety between the “open” merocyanine (spiropyran-open) and “closed” spiropyran (spiropyran-closed) forms. Employing eutectic Ga-In (EGaIn) as the top contact (Figure 9e), Kumar et al. studied a hexanethiol mixed spiropyran SAM junction and observed that the current density increased 35-fold at 1 V when the spiropyran-closed form was irradiated with UV light to induce the ring-opening reaction (Figure 9f) [123]. This was one of the highest switching ratios reported for SAM junctions. DFT calculations suggested that the density of states (DOS) was localized on the Au bottom electrode in the closed form isomer, but that it was delocalized in the open form isomer. Simulated transmission spectra validated that this delocalization not only shifted the transmission resonance closer to the gold Fermi level but also broadened it, and therefore, leading to a higher conductivity in spiropyran-open isomers.

**Figure 10:**
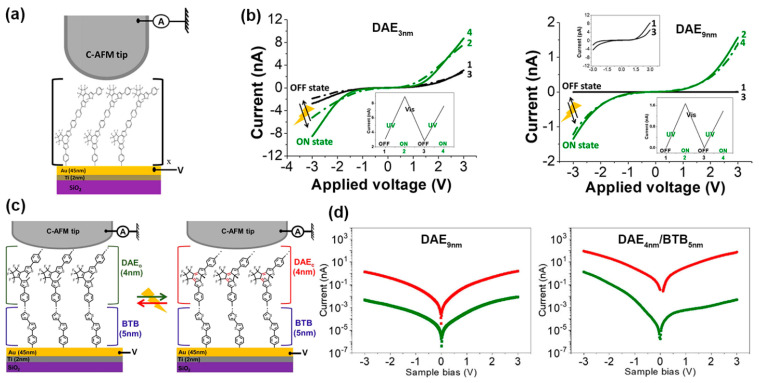
(**a**) Schematic of a SiO_2_/Ti_2 nm_/Au_45 nm_/diarylethene (DAE)_x nm_/C-AFM tip MJ; x is the diarylethene oligomer layer thickness. (**b**) I-V characteristics before (black) and after (green) UV irradiation of the DAE_3 nm_ (**left** panel) and DAE_9 nm_ (**right** panel) junctions measured by C-AFM. The upper inset shows the zoom of the black curves. Lower insets show reversibility. Reprinted with permission from ref. [124]. Copyright 2020, American Chemical Society. (**c**) Schematic of a SiO_2_/Ti_2_ nm/Au_45 nm_/BTB/DAE/C-AFM tip MJ and the photoswitching of the diarylethene oligomer units between UV and visible light. (**d**) Current in log scale versus bias characteristics before (green) and after (red) UV irradiation of DAE_9 nm_ (**left** panel) and DAE_4 nm_/BTB_5 nm_ (**right** panel) junctions measured by C-AFM. Reprinted with permission from ref. [125]. Copyright 2021, American Chemical Society.

Imen et al. prepared thin molecular layers of photochromic diarylethene (DAE) oligomers, which were deposited by an electrochemical reduction of a diazonium salt on glassy carbon and gold electrodes [124]. A metal-coated AFM tip was used as the top contact electrode (Figure 10a). MJs were constructed with diarylethene oligomer layer thicknesses of 2–3 nm and 8–9 nm, i.e., below and above the direct tunneling limit. For both junctions, the initial diarylethene layers were in their open form (OFF state with lower conductance). Upon UV irradiation at 365 nm, both junctions were switched to the diarylethene, namely the closed form (ON state with higher conductance). The two junctions were then switched back to the initial state when visible light irradiation at 435 nm was used. It is demonstrated that both layers can be reversibly switched. Interestingly, a very low ON/OFF ratio of 2~3 was observed in the direct tunneling regime for 3 nm thick diarylethene MJs. In comparison, an ON/OFF ratio of 200~400 was reproducibly obtained in the hopping regime for 9 nm thick diarylethene MJs (Figure 10b). To further enhance the ON/OFF ratio, Imen et al. fabricated a 9 nm thick bilayer junction containing a 5 nm thick bisthienylbenzene (BTB) oligomers layer and a 4 nm thick diarylethene oligomer layer in another work (Figure 10c) [125]. The dominant transport mechanism is hopping as the layer thickness exceeds 5 nm. It was found that the ON/OFF ratio reached an unprecedented value of 10^4^ for the diarylethene/bisthienylbenzene bilayer system. As a comparison, the MJ with a 9 nm thick diarylethene single layer displayed a relatively low ON/OFF ratio of 200~400 (Figure 10d). The ON state of the diarylethene/bisthienylbenzene bilayer junction is much more conductive than that of the diarylethene (9 nm) single-layer junction, although both junctions have similar conductance in their OFF states. It is hypothesized that the high conductance of the diarylethene/bisthienylbenzene junction in the ON state originates from the fact that the HOMO levels of the closed form of diarylethene and bisthienylbenzene are in resonance. Additionally, bisthienylbenzene spacer probably limits the quenching effects of the bottom electrode.

To conclude on the light-driven charge transport in MJs, recent developments in photo-induced transport phenomena and underlying mechanisms of commonly used molecular species in both single MJ and SAM junction forms are presented. We also introduced recent advances in electroluminescent in MJs. These insights prompt novel molecular electronics designs and call for more emphasis to be put on molecule/electrode interface engineering and junction architectures as key factors for the development of molecular-scale photodetectors, memory devices, and light-emitting components.

## 3. Raman Sensing in MJs

In situ observation of geometrical and structural kinetics and dynamics of a single molecule has been a long-standing goal in chemistry and is also fundamentally critical for understanding the structure–property relations and transport mechanisms of molecules in electronic devices. To gain deeper insights into the influence of the molecular core and how it bonds to electrodes on electrical properties, researchers have adopted powerful optical detection techniques, such as Raman spectroscopy, to acquire bond vibrational fingerprints of MJs and further obtain electrical and spectroscopical information simultaneously. This new approach has offered unique opportunities to understand molecular-level mechanisms for a wide variety of molecular species. In this section, we discuss recent experimental advances in revealing the chemical information of MJ devices via integrating Raman spectroscopy into molecular junction platforms.

**Figure 11:**
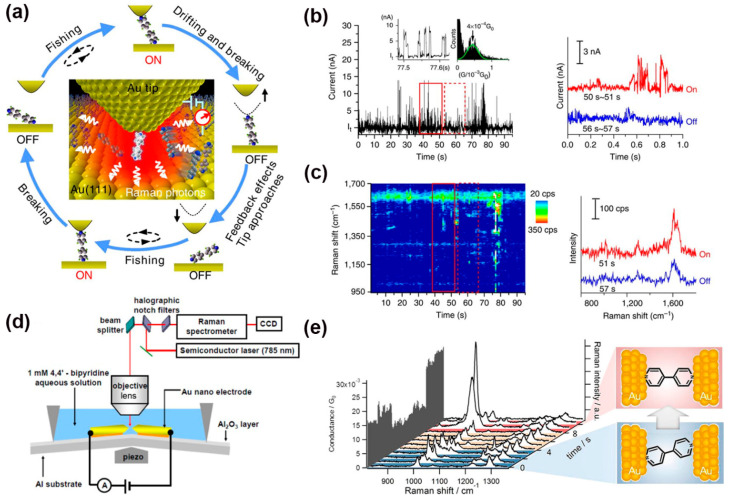
(**a**) A schematic illustration of the ‘fishing-mode’ TERS. (**b**,**c**) Simultaneous conductance and TERS measurement of 4bipy by ‘fishing-mode’ TERS. Top: Conductance versus time; bottom: TERS spectra versus time. Reprinted with permission from ref. [126]. Copyright 2011, Nature Publishing Group. (**d**) Schematic of the MCBJ-SERS measurement system. (**e**) Raman spectra and corresponding single 4,4′-bipyridine MJ geometrical structures. Reprinted with permission from ref. [127]. Copyright 2012, American Chemical Society.

Liu et al. introduced a new approach called “fishing-mode” tip-enhanced Raman spectroscopy (FM-TERS) in which molecule-to-surface bonding can be characterized during electron transport (Figure 11a) [126]. This technique enables mutually verifiable single-molecule conductance and Raman signals to be acquired simultaneously at room temperature. The working principle of “fishing-mode” TERS was as follows: the STM tip was first brought into contact with the molecule adsorbed on the gold (111) substrate to form a molecular junction; then the feedback response (i.e., the piezoresponse) of the STM was decreased by lowering the gain to continuously record conductance. The TERS junction broke and reformed spontaneously under thermal or mechanical drift. The TERS spectra and the conductance dynamics of 4bipy were acquired at the same time by FM-TERS (Figure 11b,c). This approach has been demonstrated to be a highly efficient and simple one for investigating the relationships between structural, electrical, and optical properties of various materials. Besides, simultaneous measurements of conductance and SERS signals were also carried out on a single 4,4′-bipyridine MJ in solution at room temperature using the MCBJ technique (Figure 11d) [127]. The motion of 4,4′-bipyridine between vertical and tilted configurations in the gold nanogap was identified through the observation of mode switching between b1 and b2 modes in SERS. The results confirmed that a slight increase in the tilting angle of the molecule causes an increase in the energy of Raman modes, which leads to a decrease in the conductance of the MJ (Figure 11e).

In addition to the TERS-STM-BJ and MCBJ-SERS platforms, Jeong et al. designed and fabricated a MEMS-based break junction (MEMS-BJ) device, the heart of which is a Si microelectromechanical-system (MEMS) chip [128]. This MEMS-BJ platform can also perform on-chip electrical, optical, and mechanical characterizations of MJs. Integrating real-time Raman spectroscopy and molecular conductance measurements along with the mechanical modulation, their experimental results revealed a clear interplay between the molecular structure and the electron transport. The same team further demonstrated that this on-chip platform coupled with Raman spectroscopy can provide subnanometer mechanical resolution over a wide temperature range, which may pave the way to achieving high-throughput electrical characterization of single-molecule devices [129].

**Figure 12:**
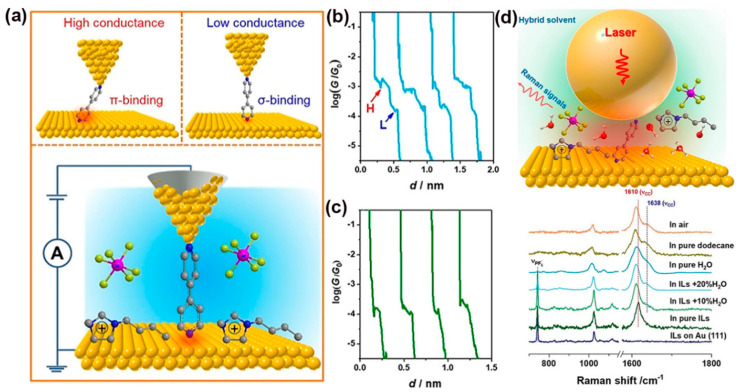
(**a**) Schematic illustration of two different 4,4′-BPY configurations (upper panel) during STM-BJ measurement (lower panel). (**b**,**c**) Representative conductance-distance traces of 4,4′-BPY in aqueous solutions(**b**) and the BMIPF_6_ (**c**). (**d**) Schematic diagram of SHINERS for probing molecular adsorption on Au (111) surfaces (top panel). SHINERS spectra of 4,4′-BPY adsorbed on Au (111) in diffident solvent environments (bottom panel). Reprinted with permission from ref. [130]. Copyright 2021, WILEY-VCH Verlag GmbH & Co. KGaA, Weinheim.

However, a potential drawback of the techniques introduced above is that the total Raman scattering signal from the tip area is rather weak. To overcome this issue, the shell isolated nanoparticle-enhanced Raman spectroscopy (SHINERS) technique was invented and used in MJ studies. Recently, Yu et al. combined the STM-BJ technique and SHINERS to probe a solvent-induced interfacial electronic effect (i.e., the interfacial electronic effect on the adsorption configuration and electronic interaction of molecules on Au substrates caused by simple solvent modification) for tuning the electron transport and adsorption geometries of the 4,4′-bipyridine (4,4′-BPY) single-molecule on the Au (111) surface [130]. As shown in Figure 12a, 4,4′-BPY adsorptions on Au (111) through the σ-bond of its N atom (σ-binding) or the π-bond of its aromatic ring (π-binding) led to two obvious conductance states in the molecular junction. The STM-BJ measurements showed that there were two distinct sets of conductance values for 4,4′-BPY in an aqueous solution near 10^−3.0^ G_0_ (H-high conductance) and 10^−3.8^ G_0_ (L-low conductance) (Figure 12b). A similar feature was observed for 4,4′-BPY in the atmosphere and in dodecane. Interestingly, there was only one set of conductance around 10^−3.9^ G_0_ for 4,4′-BPY in 1-butyl-3-methylimidazolium cations -containing ionic liquids (BMIPF_6_) (Figure 12c). Conductance measurements revealed that modifying the Au (111) surface with BMIPF_6_ could effectively stabilize the adsorption of 4,4′-BPY in a vertical/tilted orientation with σ-binding rather than flat π-binding when exposed to air, aqueous solutions, and dodecane environments. What is more, in situ SHINERS was used to offer direct spectroscopic evidence of adsorption geometry modulation at the Au (111) surface. A 55 nm Au core and 2 nm thick SiO_2_ shell nanoparticles (SHINs) were prepared first, then 4,4′-BPY SAMs were drop-casted onto the Au (111) surface (Figure 12d). In such a setup, Raman signals can be enhanced by 7~8 orders of magnitude in the nanogap between the SHINs and Au (111). It was demonstrated that a small new band at 1638 cm^−1^ appeared in the hybrid solvents (water added to BMIPF_6_ with different volume ratios) above 10% $V_{H_{2}O}$, air, aqueous solution, and dodecane (Figure 12d). This splitting of the 1610 cm^−1^ band was originating from the strong interaction between the pyridyl ring and the Au substrate and can be ascribed to the flat orientation. Thus, combining both SHINERS and STM-BJ was a good way to probe the interfacial effects on molecular adsorption at atomically flat surfaces. Zhang et al. adopted SHINERS to study the adsorption and photo-induced behavior of dye (N719) molecules on different TiO_2_ (hkl) surfaces [131]. Thiocyanate was revealed to be the main adsorption group of the N719 dye molecule at different rutile TiO_2_(hkl) surfaces and plays a determining role in its stability. Additionally, it was indicated that N719 molecules exhibited long-term stability when adsorbed on TiO_2_ (111), which will be helpful for the rational design of dye-sensitized solar cells.

**Figure 13:**
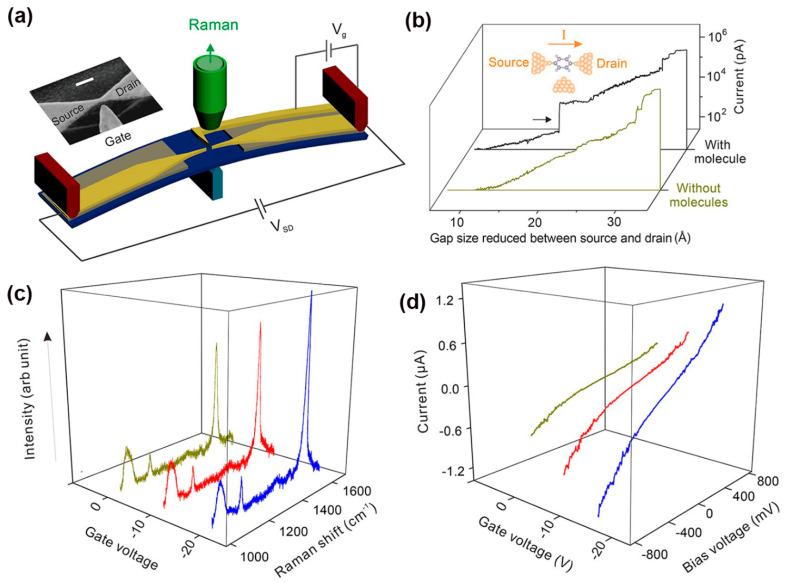
(**a**) Experimental schematic of side-gating Raman scattering. Scale bar is 100 nm for the inset SEM image of the MCBJ chip with a side-gate electrode. (**b**) Current curves when two electrodes approach with and without assembled molecules. (**c**) SERS spectra recorded at different gate voltages and (**d**) I-V curves of 1,4-benzenedithiol MJ upon different gate voltages. Reprinted with permission from ref. [132]. Copyright 2018, American Chemical Society.

To further enhance the sensitivity of Raman scattering, other factors that modulate the Raman response have been investigated. For example, Guo et al. recently employed a modified MCBJ technique and demonstrated a single-molecule based field-effect Raman scattering device in two-terminal MJs coupled with an additional third electrode as gate [132]. The junction structure is shown in Figure 13a. The side-gate electrode perpendicular to the source-drain electrodes did not undergo any stretching force during the substrate bending process in MCBJ measurements. As shown in Figure 13b, the current jump indicated the formation of a single MJ, while no current jump was observed when the molecule was absent. Due to the gating effect, the Raman intensity of 1,4-benzenedithiol in the junction increased dramatically as the gate voltage switched from 0 to -20 V (Figure 13c). Figure 13d displays the I-V characteristics of a single-molecular junction as a function of the gate voltage. It was observed that the junction conductance increased as the gating voltage changed to more negative values. Both measurements and DFT calculations supported that Raman scattering intensity can be enhanced by 40% beyond the limit of electromagnetic enhancement via the electrostatic gating of molecular orbitals.

In this section, we have presented several emerging techniques that combined Raman spectroscopy with MJ techniques, including TERS, MCBJ-SERS, MEMSBJ-SERS, and SHINERS. These experimental tools have been proven to be capable of directly monitoring conformational change, chemical reaction, and molecule/electrode interface coupling in MJ systems, which were inaccessible in conventional electrical measurements. Beyond the common two-terminal layout, molecular orbital gating has also been shown to effectively enhance the Raman scattering intensity. It is anticipated that these techniques will be extensively used to capture a complete picture of the molecular behavior in future MJ measurements.

## 4. Conclusions and Outlook

As a remarkably fertile platform for studying physical phenomena at the molecular scale, MJs have enabled direct investigation of light-matter interactions at the single-molecule level. The past decade has seen a rapidly growing interest in understanding how charge transport in MJs is impacted by light illumination and a variety of emergent effects associated with it. In this review, we have systematically discussed recent progress in probing the electrical and optical properties of illuminated MJs composed of a wide spectrum of molecular species in both single-molecule and SAM configurations. Not only have these recent studies proven that transport properties of MJs can be effectively modulated by optical means, but they have also demonstrated that combining optical sensing techniques, like Raman spectroscopy, with electrical measurements could vastly deepen our knowledge of the structure–property correlation of individual molecules in the junction. More excitingly, optoelectronic behaviors similar to (and even beyond) those of conventional semiconductor materials, such as photoswitching and photoemission, have also been experimentally realized in molecular-scale devices. It is evident that this new knowledge and technical advancements have laid a solid foundation for the development of future molecular optoelectronic components.

However, before molecular-scale optoelectronics become practical in our daily lives, several outstanding challenges need to be addressed. First, there still exist open questions in understanding light-driven transport in MJ devices, for example, how to isolate the effect of light-molecule interactions from other interactions involved in the junction. Second, there is a clear limitation in suitable molecular candidates. Future improvements in designing and synthesizing new molecular species with promising optoelectronic properties are required. The optoelectronic properties of more complex systems, such as supramolecules, molecular machines, and polymers, have rarely been examined. It is expected that new quantum transport phenomena, which are not possible in the bulk (or ensemble measurements), may occur in these systems. Third, discrepancies have been observed between single-molecule and SAM junctions composed of the same molecule. It is critical to uncover the origin of this inconsistency, as it is beneficial for designing large area devices via molecular assembly. Fourth, there is an urgent need to investigate emerging MJ device structures and architectures, such as incorporating plasmonic nanostructures into MJs. These new devices and measurement schemes may catalyze novel optoelectronic functionalities that were previously not envisioned. Specifically, as demonstrated in several recent studies, the integration of plasmonics with molecular junction electronics has become a promising future direction for molecular optoelectronics. Lastly, from the theoretical point of view, it has remained challenging to precisely simulate the transmission of MJs and the effect of light illumination. It is evident that the development of accurate computational tools is urgently required.

Finally, we note that breakthroughs in this multidisciplinary research area will rely on intimate collaborations from materials synthesis, instrumentation, nano-characterization, and theoretical simulation to establish the fundamental mechanisms and to develop new design principles at both the molecular and device levels. Given the remarkable advances in recent years and continued efforts by researchers in this field worldwide, there is no doubt that optoelectronics research at the molecular scale will continue to progress and achieve the transition from the research laboratory to real applications.

## Data Availability

Not Applicable.
